# Supplementary material for: Investigating Carvedilol’s Repurposing for the Treatment of Non-Small Cell Lung Cancer via Aldehyde Dehydrogenase Activity Modulation in the Presence of β-Adrenergic Agonists
Source: Curr Issues Mol Biol. 2023 Sep 29;45(10):7996–8012. doi: 10.3390/cimb45100505 (PMC10605277; doi:10.3390/cimb45100505)
Supplement: Supplementary file 1 [file cimb-45-00505-s001.zip › cimb-2611617-supplementary.pdf]

Investigating Carvedilol's Repurposing for the Treatment of Non-Small Cell Lung Cancer via Aldehyde Dehydrogenase Activity Modulation in the Presence of β-Adrenergic Agonists

Balqis A. Ikhmais [1,*], Alaa M. Hammad [1], Osama H. Abusara [1], Lama Hamadneh [2], Hamza Abumansour [1], Qasem M. Abdallah [3], Ali I. M. Ibrahim [1], Lina Elsalem [4], Mariam Awad [1] and Rahaf Alshehada [1]

[1] Department of Pharmacy, Faculty of Pharmacy, Al-Zaytoonah University of Jordan, P.O. Box 130, Amman 11733, Jordan; alaa.hammad@zuj.edu.jo (A.M.H.); o.abusara@zuj.edu.jo (O.H.A.); h.abumansour@zuj.edu.jo (H.A.); a.ibrahim@zuj.edu.jo (A.I.M.I.); mariam7asan@hotmail.com (M.A.); rahafsaidghaith@gmail.com (R.A.)

[2] Department of Basic Medical Sciences, Faculty of Medicine, Al-Balqa Applied University, P.O. Box 206, Al-Salt 19117, Jordan; lama.hamadneh@bau.edu.jo

[3] Department of Pharmacology and Biomedical Sciences, Faculty of Pharmacy and Medical Sciences, University of Petra, P.O. Box 961343, Amman 1196, Jordan; qasem.abdallah@uop.edu.jo

[4] Department of Pharmacology, Faculty of Medicine, Jordan University of Science and Technology, P.O. Box 3030, Irbid 22110, Jordan; lmelsalem@just.edu.jo

* Correspondence: b.ikhmais@zuj.edu.jo

Supplementary Material

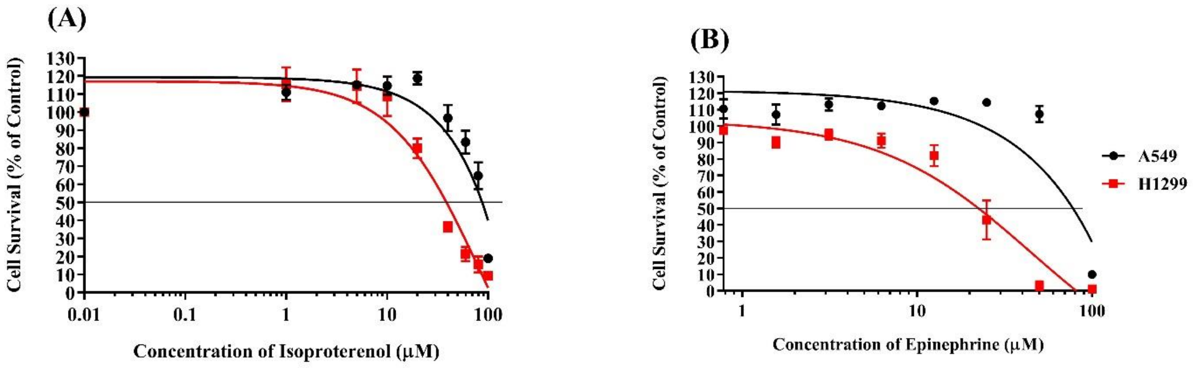

Figure S1. Dose-response curves for NSCLC cell lines; A549 and H1299 treated with β-agonists (**A**) isoproterenol and (**B**) epinephrine) as measured by the MTT assay. Treatment for 96 h; $n = 3$.

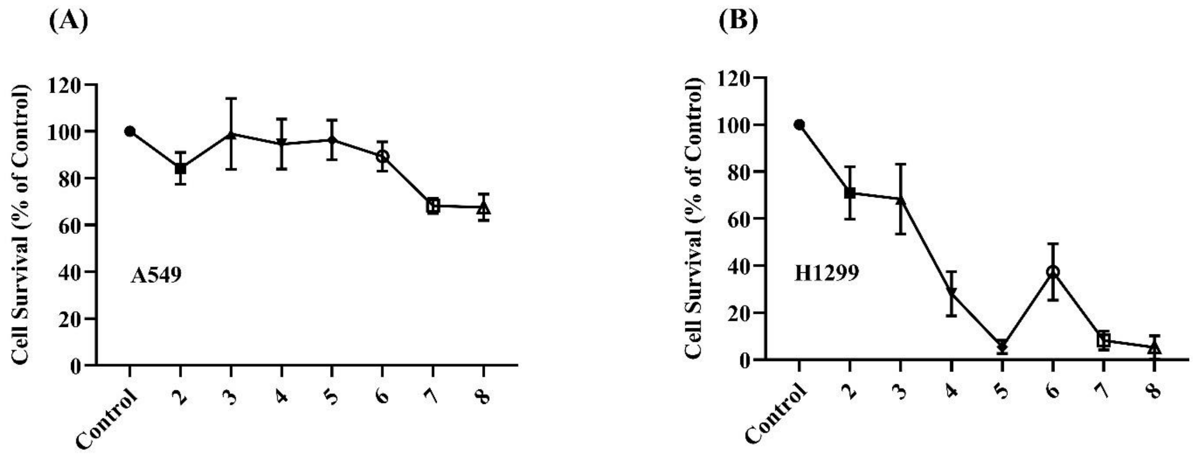

Figure S2. Cytotoxicity of different treatment conditions numbered as follow: 2, 3, 4, 5, 6, 7, and 8, which indicated A549 (curve (**A**)) and H1299 (curve (**B**)) cells treated with CAR at 10 µM, ISO at 20, 40, and 60 µM and CAR 10 µM plus ISO 20, 40 and 60 µM concentrations, respectively, as measured by MTT assay. Treatment for 96 h; $n = 3$.
